# Identification of pathological-related and diagnostic potential circular RNAs in Stanford type A aortic dissection

**DOI:** 10.3389/fcvm.2022.1074835

**Published:** 2023-01-13

**Authors:** Qiao Liang, Zeyi Zhou, Hui Li, Qing Tao, Yali Wang, Anqi Lin, Jing Xu, Bin Zhang, Yongzheng Wu, Haiyan Min, Lei Wang, Shiyu Song, Dongjin Wang, Qian Gao

**Affiliations:** ^1^Center for Translational Medicine and Jiangsu Key Laboratory of Molecular Medicine, Medical School of Nanjing University, Nanjing, Jiangsu, China; ^2^Department of Thoracic and Cardiovascular Surgery, Institute of Cardiothoracic Vascular Disease, Nanjing University, Affiliated Drum Tower Hospital of Nanjing University Medical School, Nanjing, Jiangsu, China; ^3^Central Laboratory, Nanjing Chest Hospital, Nanjing Medical University, Nanjing, China; ^4^Central Laboratory, The Second Affiliated Hospital of Nanjing University of Chinese Medicine, Nanjing, China; ^5^Department of Clinical Laboratory, Jiangsu Provincial Hospital of Integrated Chinese and Western Medicine, Jiangsu Province Academy of Traditional Chinese Medicine, Nanjing, China

**Keywords:** circular RNAs, Stanford type A aortic dissection, inflammation, extracellular matrix, immune infiltration

## Abstract

**Introduction:**

Stanford type A aortic dissection (TAAD) is one of the lethal macrovascular diseases caused by the invasion of blood into the media layer of ascending aortic wall. Inflammation, smooth muscle dysfunction, and extracellular matrix (ECM) degradation were regarded as the major pathology in affected tissue. However, the expression pattern and its regulation especially through circular RNAs (circRNAs) as an overall characteristic of TAAD molecular pathology remain unclear.

**Methods:**

We employed CIRCexplorer2 to identify circRNAs based on the RNA sequencing (RNA-seq) data of human ascending aortic tissues to systematically assess the role of circRNA in the massive alterations of gene expression in TAAD aortas. The key circRNAs were determined by LASSO model and functionally annotated by competing endogenous RNAs (ceRNA) network and co-analysis with mRNA profile. The expression level and diagnostic capability of the 4 key circRNAs in peripheral serum were confirmed by real-time polymerase chain reaction (RT-PCR).

**Results:**

The 4 key circRNAs, namely circPTGR1 (chr9:114341075-114348445[−]), circNOX4 (chr11:89069012-89106660[−]), circAMN1 (chr12:31854796-31862359[−]) and circUSP3 (chr15:63845913-63855207[+]), demonstrated a high power to discriminate between TAAD and control tissues, suggesting that these molecules stand for a major difference between the tissues at gene regulation level. Functionally, the ceRNA network of circRNA-miRNA-mRNA predicted by the online databases, combining gene set enrichment analysis (GSEA) and cell component prediction, revealed that the identified circRNAs covered all the aspects of primary TAAD pathology, centralized with increasing inflammatory factors and cells, and ECM destruction and loss of vascular inherent cells along with the circRNAs. Importantly, we validated the high concentration and diagnostic capability of the 4 key circRNAs in the peripheral serum in TAAD patients.

**Discussion:**

This study reinforces the vital status of circRNAs in TAAD and the possibility of serving as promising diagnostic biomarkers.

## Introduction

Aortic dissection is an acute aortic syndrome clinically characterized by its abrupt onset and high mortality (1, 2), which can be divided into Stanford type A (TAAD) and Stanford type B (TBAD) while the former affects the ascending aorta rather than the latter (3). Despite the continuous development of surgical treatment and medication, the mortality rate of TAAD remains high (2, 4). Thus, elucidating the molecular nature of TAAD pathology and developing effective early diagnostic and therapeutic strategies upon critical molecules are urgently desirable. Currently, the cell adhesion disorder, extracellular matrix (ECM) degradation (5), and associated inflammation caused by endothelial cell and vascular smooth muscle cell (VSMC) damages are believed to be the key link in the pathology of TAAD (6). Other biological processes, such as hypoxia and phenotype switch of smooth muscle cells, are observed by individual studies (7, 8). However, the organized senior regulatory mechanisms of this multi-event pathological process remain unestablished.

Circular RNAs (circRNAs) are a set of transcriptionally expressed and spliced RNAs composed of an enormous amount of species, each of which covalently forms a closed loop through a cis-linkage between its 5' and 3' ends with the currently unknown biological purpose (9). However, due to this unusual structure, circRNAs can resist exonuclease and maintain high stability both in the internal and external environments of cells, with a nearly 5 times longer half-life (48 vs. 10 h) compared to their parental mRNAs (10). The expression pattern of circRNA is believed to be tissue-specific and cell-specific and has no obvious correlation with their host gene expression (9). They can participate in normal as well as pathological processes through numerous mechanisms, including competing with linear splicing, acting as miRNA sponges, binding to mRNA-related proteins, and regulating gene expressions at the epigenetic level (11–13). The high stability and powerful gene-expression regulation ability of circRNAs place themselves in a superior status of complicated biological and pathological processes.

Growing evidence has implicated circRNAs in various diseases, such as diabetes mellitus, neurological disorders, cardiovascular diseases, and cancers (9). For example, circANRIL, a circRNA located at 9p21, a locus known to be involved in atherosclerosis, can bind to pescadillo homolog 1 (PES1) and restrain 60S-preribosomal assembly in VSMCs and macrophages, which lead to cell apoptosis and atherosclerosis aggravation (14). Moreover, the environmental constancy and temporal and spatial specificity of circRNAs' expression endow them the diagnostic potentiality.

However, few studies have focused on the circRNAs in the pathogenesis of TAAD, with the exception of circMARK3 and hsa_circRNA_101238, which were reported upregulated in TAAD (15, 16). A systematic and optimized analysis of circRNAs in this context was not performed, leaving the insecureness. In this study, we applied RNA-seq analysis to investigate circRNA profiles and identify the differentially expressed (DE) circRNAs in TAAD as the key differentiator. The function of these key circRNAs was predicted by the construction of the circRNA-miRNA-mRNA network as well as correlation analysis of circRNAs and mRNAs in the present dataset. We further established the relationship of the levels of the key circRNAs with the cell compositions by xCell analysis, which characterized the pathology in TAAD aortic tissue, and accessed the diagnostic probability of the circRNAs in the peripheral serum in TAAD by RT-PCR.

## Materials and methods

### Clinical specimens

This study was conducted in accordance with the Declaration of Helsinki and was approved by the Medical Ethics Committee of Nanjing Drum Tower Hospital, the affiliated hospital of Nanjing University Medical School (Institutional Review Board File 2016-152-01). Written informed consent was collected from all subjects. The venous blood samples of diseased participants, including 8 patients with TAAD, 9 patients with TBAD, and 4 patients with intramural aortic hematoma (IAH), and 10 age-matched normal controls (NC) were obtained from Nanjing Drum Tower Hospital and diagnosed by contrast-enhanced CT. The specimens were immediately processed after collection. Blood samples were centrifuged at 2,000 rpm for 10 min at 4°C, and the supernatant was transferred into RNase-free tubes, which were subsequently stored at −80°C. The baseline information of enrolled samples was listed as follows.

**Table:** 

	**NC (*****N*** = **10)**	**TAAD (*****N*** = **8)**	**TBAD (*****N*** = **9)**	**IAH (*****N*** = **4)**	**Overall (*****N*** = **31)**
**Sex**					
F	4 (40.0%)	4 (50.0%)	4 (44.6%)	2 (50.0%)	14 (45.2%)
M	6 (60.0%)	4 (50.0%)	5 (55.6%)	2 (50.0%)	17 (54.8%)
**Age**					
Mean (SD)	47.6 (7.01)	49.8 (8.21)	51.8 (14.2)	51.3 (7.89)	49.8 (9.67)
Median [Min, Max]	47.5 [37.0, 62.0]	50.5 [34.0, 59.0]	52.0 [35.0, 76.0]	53.5 [40.0,58.0]	50.0 [34.0,76.0]

### Quantitative real-time PCR

Total RNA was isolated from cells using miRNeasy Serum/Plasma Kit (QIAGEN, Hilden, Germany) and was reverse transcribed using iScript cDNA Synthesis Kit (Vazyme, Nanjing, China). RT-PCR was performed with SYBR green PCR Master Mix (Vazyme, Nanjing, China) using ViiA 7 Real-Time PCR System (Applied Biosystems, Waltham, CA). The primers used in this work were listed in Table 1. In each qPCR experiment, the same samples were performed in triplicate, and the same experiment was performed at least three times. The quantification of the qPCR results was achieved by a comparative method 2^−ΔΔCT^ using β-actin as an internal control (the calibrator) from the respective control serum.

**Table 1:** Primers of humans used for real-time quantitative PCR analysis.

**Gene**	**Forward primer**	**Reverse primer**
β-Actin	GGACGTACAACTGGTATTGTGC	TCGGCAGTAGTCACGAAGGA
circAMN1	GCTCTCCTGCACCTGTCTAA	CTGTCTTTTATGTTGGGAGGCA
circNOX4	TGGCAAGAGAACAGACCTGA	GAAGGGCAGAATTTCGGAGTC
circUSP3	CGCCGTGGAGTTAAGGAATG	ACCACAAAATCATCACAGCGA
circPTGR1	CTCAAGGGCTGCAAAGTTGT	AGAAATGGAGTGCGTTGTCC

### Data collection and preprocessing

In this study, we analyzed RNA-seq data from 20 samples of 10 patients with TAAD and 10 healthy controls produced in a previous study. The dataset GSE153434, including transcriptome expression and sequence read archive (SRA) files, was downloaded from the Gene Expression Omnibus (GEO) database (https://www.ncbi.nlm.nih.gov/geo). The protein-coding genes in transcriptome expression were employed for identifying DEGs by DEseq2 (version 1.34.0) with the threshold of *p* < 0.05 and |log2 fold change (FC)|>1.

### CircRNAs identification and differential expression

After implementing the initial quality assessment, filtered data were mapped to the human reference genome (GRCh37/hg19) with STAR (version 2.7.10a) (17). CIRCexplorer2 (version 2.3.8) was utilized to predict circRNAs based on the gencode.v19.annotation.gtf (13). Raw back splice-junction (BSJ)-spanning reads were analyzed using DEseq2 differential expression analysis (using a *p* < 0.05 and |log2FC|>1) to identify differentially expressed between the different sample groups.

### Construction of circRNA lasso model of TAAD

A 10-fold cross-validation lasso regression model was implemented in the R package glmnet (version 4.1–4) according to a binomial response type for binary dependent variables (18). The largest value of lambda which makes the error within 1 standard error of the minimum is utilized in this model. The receiver operating characteristic (ROC) curve was drawn by the R package ROCR (version 1.0–11) (19).

### Prediction of circRNA-miRNA-mRNA interactions

CircRNA–miRNA interaction prediction was based on circBank, a comprehensive database of human circRNA which contained over 140,000 human-annotated circRNAs (20). The information on miRNA–mRNA regulatory relationships was identified by miRNAtap (version 1.28.0), an R package that allows to integrate ranked miRNA target predictions from multiple sources available online. mRNAs predicted at least two times among the five online sources, including PicTar, DIANA, TargetScan, miRanda, and miRDB, were considered as target candidates of certain miRNAs. The shared genes of TAAD overexpressed genes and miRNAs target genes were considered as the candidate targets of circRNA-miRNA-mRNA interactions.

### Gene functional enrichment analysis

The module gene enrichment analysis was carried out by the R package clusterProfiler (version 4.2.2) (21). Gene Ontology (GO), including biological process (BP), cellular components (CC), molecular function (MF), and Kyoto Encyclopedia of Genes and Genomes (KEGG) were analyzed. The *p* < 0.05 was identified as a significant outcome.

### Gene set enrichment analysis

For the identification of enriched gene signatures, we applied the gene set enrichment analysis (GSEA) based on R package clusterProfiler (21). The gene sets of HALLMARK, KEGG, and REACTOME were downloaded from the Broad Institute website (http://www.gsea-msigdb.org/gsea/index.jsp).

Gene set enrichment analysis was performed by comparison of gene-expression data obtained from TAAD and NC, as well as Spearman's correlation of circRNAs with gene-expression data. We used 1,000 gene-set permutations for testing of significance. The default parameters were used in this section.

### Calculation of cell infiltration

We used xCell (version 1.1.0) to calculate the relative proportion of 64 infiltrating cells in each sample based on the gene-expression profile. The different score of infiltrating cells was determined according to Student's *t*-test after the normal distribution test.

### Statistical analyses

R (version 4.1.2) was applied to all statistical tests. Spearman's correlation analyses were done by R. The multiple *T*-test was adopted to compare the differences of expression of indicated circRNAs between the TAAD, TBAD and IAH groups and the NC group, respectively. We did the normal distribution test of circRNA relative expression by R function “shapiro.test.” All *p*-values were considered significant if < 0.05.

## Results

### Functional DEGs profile highly resembles the molecular pathology of TAAD

**Figure 1:**
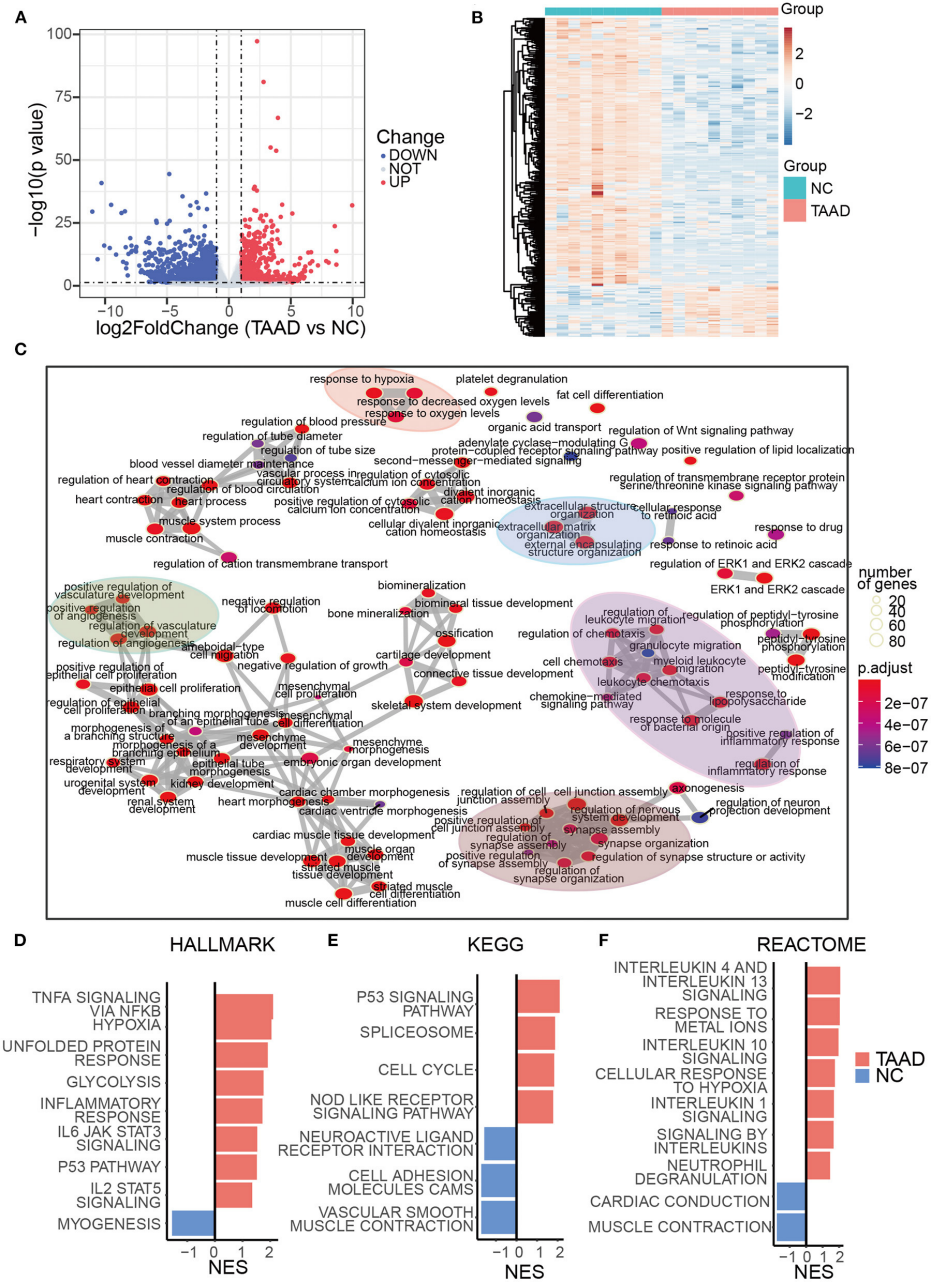
Transcriptome signatures of TAAD aorta were highly related to elevated inflammation, loss of mesenchyme, and ECM reconstruction. **(A)** Volcano plots of DEGs. The blue and red parts indicate >2-fold decreased and increased expression of the dysregulated mRNAs in TAAD tissues, respectively (*P* < 0.05). **(B)** Heatmap represents TAAD DEGs. **(C)** DEGs enrichment map of enriched terms into a network with edges connecting overlapping gene sets. **(D–F)** GSEA of HALLMARK **(D)**, KEGG **(E)**, and REACTOME **(F)** gene sets from the Molecular Signatures Database of the Broad Institute, showing the most significantly enriched gene sets in TAAD and normal control aortic tissues and their normalized enrichment scores (NES).

We first utilized the expression matrices of the GSE153434 dataset to assess the TAAD molecular pathology. Under the threshold of *p* < 0.05 and |log2FC| > 1, a total of 1,874 DEGs, with 643 upregulated DEGs and 1,231 downregulated DEGs in the aortas of patients with TAAD, were identified (Figures 1A, B). Consistent with previous studies, the enrichment analysis of DEGs was focused on the extracellular matrix reorganization and inflammation (Figure 1C), which contributes to vascular remodeling and weakening of the aortic wall (22, 23). Specifically, the expressions of collagen, COL9A3 and COL6A6, and fibrillin (FBN2) were significantly downregulated in TAAD, while the previously reported TAAD biomarkers COL3A1 and FBN1 (24, 25) were only slightly reduced in TAAD, whose log2FC was 0.72 and 0.60, respectively (Supplementary Table 1). Metalloproteinases (MMPs) also exhibited distinct expression patterns with MMP1, 3, 10, 14, and 19 overexpressed, which were able to degrade collagen, fibronectin, laminin, and gelatins and were mainly produced by fibroblasts and smooth muscle cells (26), while MMP15, 16, 23B, and 28 underexpressed in TAAD. Additionally, tissue inhibitors of metalloproteinases (TIMPs) demonstrated similar expression patterns with previous studies (27, 28), with TIMP1 upregulated and TIMP3 downregulated in TAAD. While both were capable of inhibiting all known MMPs (29, 30), TIMP1 had a cytokine-like function and angiogenesis-suppressing effect, which was MMP-independent (31), explaining the potential significance of this expression pattern shift.

Moreover, the hypoxia-related response was also enriched in the present dataset, which was regarded as an exacerbation factor of aortic dissection by reprogramming macrophage (32). Interestingly, the various items regarding the transport, signaling, and homeostasis of calcium ions were all well observed. The genes responsible for calcium ion transport into the cytosol, e.g., RAMP3, GRIN2A, PKD2, PLN, SLC8A1, APLNR, CLIC2, and CASQ2, were all downregulated (Supplementary Table 1), which likely reflected the pathology of VSMCs that indeed was suggested in aged patients with TAAD (33).

As for inflammation composition, the interleukins, IL-6 and IL-33, were overexpressed in TAAD aortas, whose log2FC was 2.86 and 1.16, respectively. IL-6 was reported to be related to the severity of aortic dissection and the time of presentation (34). IL-33, which is induced by mechanical stress and inflammatory cytokines, acted as a driver of endothelial inflammation (35). The other reported TAAD-related inflammatory factors, including TNF-α, IFNs, and IL-1, were not differentially expressed between TAAD and normal controls.

To retrieve an overall change profile of biological processes, we then employed gene set enrichment analysis (GSEA) based on all ranked log2FC values, which might benefit the assessment of enriched functions by ranking their expression fold changes. Interestingly, the GSEA revealed a broad and complicated inflammatory response, containing TNF-α, IL-1, 4, 6, 10, and 13 signaling pathways (Figures 1D, F), although most expression levels of these individual cytokines exhibited no significant difference between TAAD and normal controls. This result demonstrated a distinct activation pattern of inflammation at the levels of both upstream and downstream components for the same signaling pathways and increased the awareness of the potential uniqueness of TAAD inflammation. We also found that the weakening of the aortic wall (VSMCs contraction and cell adhesion cams) was highly enriched in TAAD aortic tissue (Figures 1D–F). The loss of neuroactive ligand–receptor interaction was augmented in the TAAD group, indicating the weakening of the aortic wall and/or deprivation of its neurotrophy. Thus, a massive and complicated molecular pathology of TAAD with no regulatory hierarchy was displayed.

### CircRNA expression profile in TAAD

**Figure 2:**
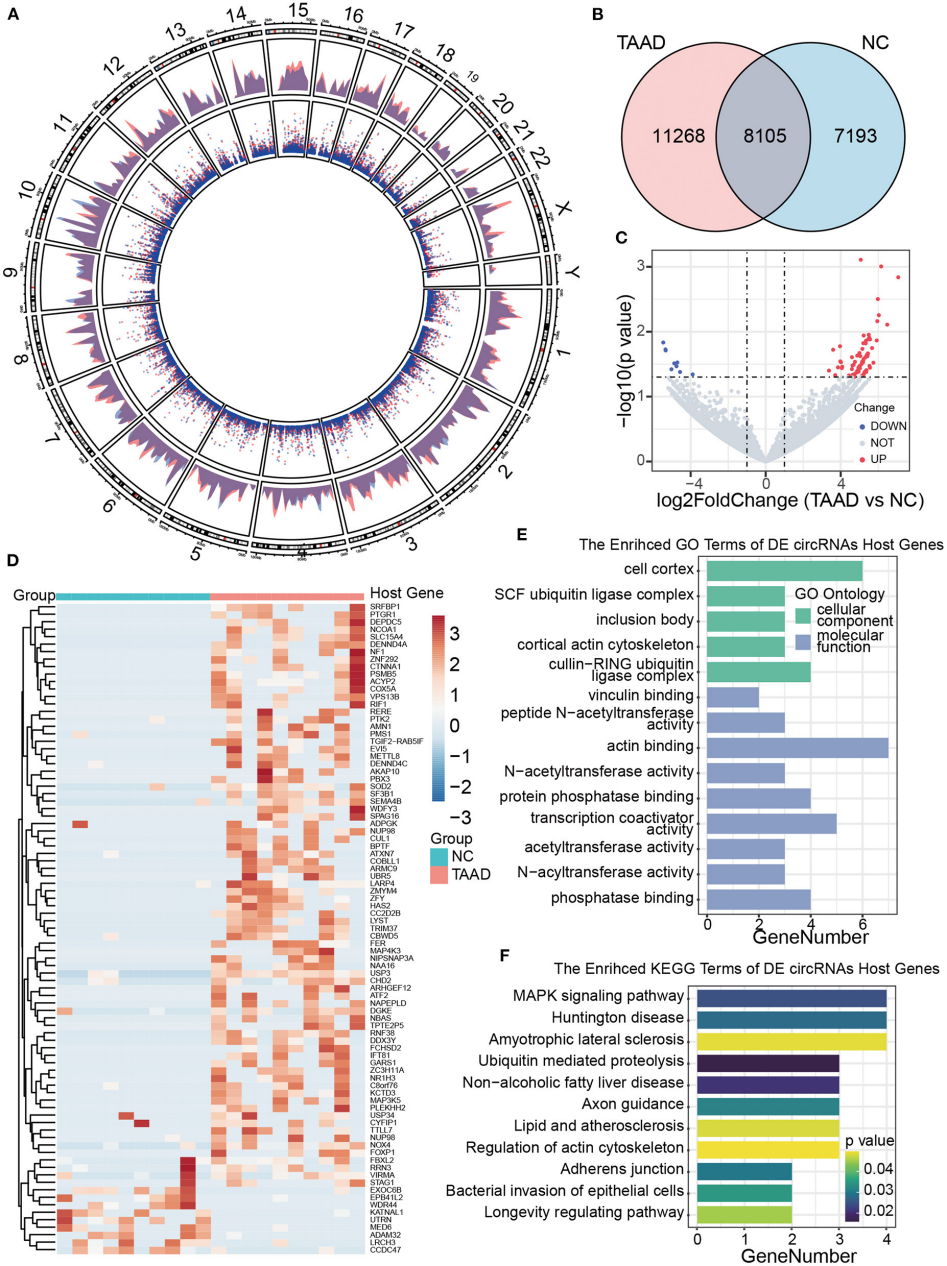
Profiles of circRNAs of patients with TAAD and healthy controls. **(A)** Distribution of the circRNAs on human chromosomes. A circos plot diagram shows the locations of the circRNAs on human chromosomes. The outer and inner layers indicate the distribution and counts of circRNAs, respectively. Red and blue represent the patients and normal controls, respectively. **(B)** Venn diagram analysis presents the number of unique and common circRNAs found in patients with TAAD and normal controls. **(C)** Volcano plot displays dysregulated circRNAs between patients with TAAD and normal controls. The horizontal black dashed line represents a *p*-value of 0.05. The gray points represent circRNAs with no significant difference between TAAD and normal individuals. The blue and red points represent significantly downregulated and upregulated circRNAs in patients with TAAD, respectively. **(D)** Hierarchical clustering of significant DE circRNAs in patients with TAAD and normal controls. The host genes of DE circRNAs were annotated on the right side. **(E, F)** The bar plots of circRNA host genes GO annotation **(E)** and KEGG **(F)** enrichment results.

To explore the regulatory mechanisms of molecular pathology in TAAD, we computed circRNAs by RNA-seq data of TAAD and normal control ascending aortas, which was downloaded from the GEO database. A total of 26,566 kinds of circRNAs were identified by CIRCexplorer2 (13), while only approximately 30% (8105) of total circRNAs were shared between TAAD and normal control (Figure 2B), indicating the existence of a major body of disease-dependent circRNAs, which located dispersedly across all human chromosomes (Figure 2A).

We next identified the DE circRNAs of the patients with TAAD and healthy controls under the threshold of *p* < 0.05 and |log2 FC|>1. Compared with healthy controls, 78 upregulated and 9 downregulated circRNAs were recognized in TAAD aortas (Figure 2C). Patients with TAAD and normal controls were diacritical according to the DE circRNAs hierarchical clustering analysis (Figure 2D; Supplementary Table 2). The GO annotation of the DE circRNAs host genes was focused on the ubiquitin, acetyltransferase, and phosphatase-related function, while the significant KEGG pathways were MAPK signaling pathway, ubiquitin-mediated proteolysis, and adherens junctions (Figures 2E, F). However, further examining the expression levels of the host genes of DE circRNAs revealed no differences in mRNA profile in TAAD, implicating no significant involvement of the host genes in TAAD, which is consistent with the previous observation (9).

### Identification of key CircRNAs of TAAD

**Figure 3:**
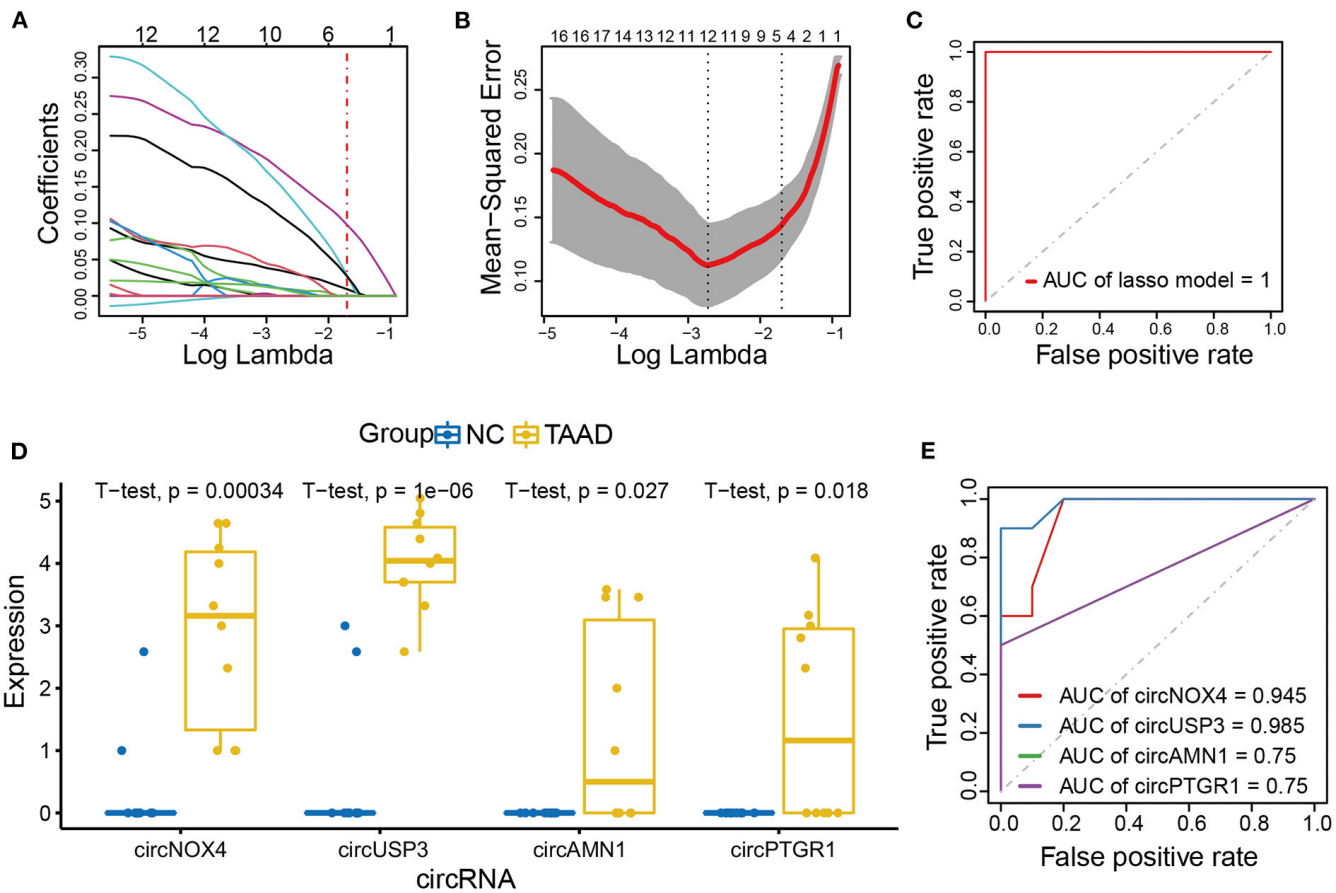
CircNOX4, circUSP3, circAMN1, and circPTGR1 were the key circRNAs of TAAD. **(A, B)** Risk score model construction for DE circRNAs using lasso regression analysis. **(A)** The lasso coefficient profiles of DE circRNAs in TAAD. **(B)** Partial likelihood deviance was plotted against log (lambda). The vertical dotted lines indicate the lambda value with minimum error and with one standard error (SE) of the minimum. **(C)** ROC curve analysis of the prognostic prediction efficiency of the lasso model. **(D)** The boxplot of lasso model circRNAs expression. **(E)** ROC curve analysis of the prognostic prediction efficiency of individual circRNA in the lasso model.

We further applied the lasso model to filter out the key circRNAs that distinguish TAAD and normal control and, therefore, might serve as biomarkers for TAAD (Figure 3A). To select the tuning parameter lambda of penalties, we employed 10-fold cross-validation to obtain the optimal lambda that makes misclassification error within 1 standard error (Figure 3B). The ROC curve was constructed to assess the diagnostic value of the lasso model in TAAD, and the AUC was 1 (Figure 3C), evidencing the diagnostic probability of these circRNAs. The lasso model comprised four key circRNAs, including circPTGR1, circNOX4, circAMN1, and circUSP3, which were all significantly increased in TAAD (Figure 3D), and individually each of them exhibited a powerful diagnostic capability ranging from 0.75 to 0.985 (Figure 3E).

### Function analysis of key circRNAs

**Figure 4:**
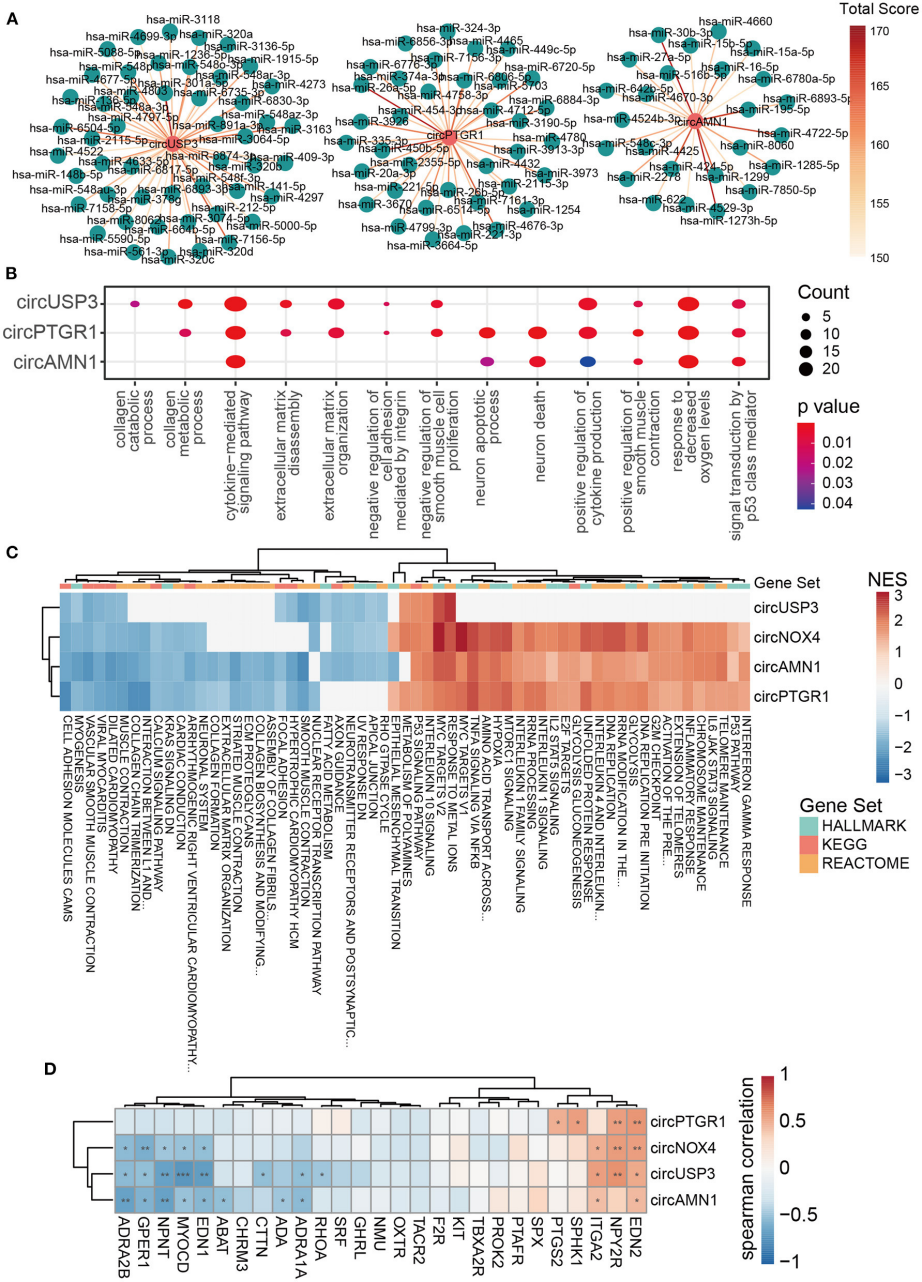
The key circRNAs of TAAD were functional in inflammation, ECM remodeling, and VSMC dysfunction. **(A)** CircRNA–miRNA interaction network predicted by circBank. The higher the total score means the greater probability that the predicted miRNA is a real one. **(B)** Dot plot of representative GO terms of miRNA target genes. The dot size and color represent the gene number and p-value of the corresponding term, respectively. **(C)** Heatmap of GSEA NES based on the correlation coefficient of circRNAs and gene expression. HALLMARK, KEGG, and REACTOME gene sets were utilized. **(D)** Heatmap of Spearman's correlation coefficient of the key circRNAs with genes involved in positive regulation of smooth muscle contraction (GO:0045987). The red rectangles indicate the target genes of circRNA-interacted miRNA. *p < 0.05, **p < 0.01, ***p < 0.001 (Spearman's correlation).

To functionally interpret the role of the key circRNAs, we next built the ceRNA network of circRNA-miRNA-mRNA based on the online databases that contained various sources of different disease-condition and tissue- and cell-specificity to model the first-order approximation of direct regulation of the expression by circRNAs in TAAD. Three of the four key circRNAs were annotated in circBank, and 36, 25, and 49 targeted miRNA candidates were filtered for circPTGR1, circAMN1, and circUSP3, respectively (Figure 4A; Supplementary Table 3). We then applied miRNAtap, which contained five online databases of PicTar (36), DIANA (37), TargetScan (38), miRanda (39), and miRDB (40), to filtrate the candidate target genes of miRNAs. The genes that were predicted two times among the five online sources were incorporated. The functions of the key circRNAs were expounded on the ground of the enrichment of the intersection of TAAD overexpressed genes and the circRNA-sponged miRNAs' target genes. The GO enrichment analysis focused on the classical pathological processes of TAAD including inflammation, ECM degradation, and negative regulation of smooth muscle cell proliferation (Figure 4B), indicating that the circRNA-miRNA-mRNA network of the key circRNAs was sufficient in TAAD pathology. Additionally, responses to decreased oxygen levels, neuron death, P53 signal transduction, and cell adhesion weakening were also found, which were all consistent with DEGs enrichment analysis (Figure 4B).

To cross-examine the above findings, we then applied GSEA based on the correlation of circRNAs and mRNA expression profile. The HALLMARK, KEGG, and REACTOME gene sets of the Broad Institute website were utilized. The key circRNAs correlated terms covered the majority of GSEA results of DEGs, with 87% (61/70) enriched terms and 89% (16/18) repressed terms (data not shown). Impressively, the function of the key circRNAs was almost identical in three gene sets and was accordant with the target genes enrichment in ceRNA, likely implicating the domination of the circRNA-sponged miRNA function in the TAAD context. The inflammatory response (IFN-γ, TNF-α, IL-1, IL-2, IL-4, IL-6, IL-10, IL-13 signaling), P53 signaling pathway, and hypoxia were enriched by increasing the key circRNAs, while the ECM components (collagen chain trimerization, collagen biosynthesis and modifying, ECM proteoglycans, ECM organization) and cell adhesion (cell adhesion molecules cams and apical junction) were enriched by decreasing the key circRNAs (Figure 4C). As for the relationship of the circRNAs with ECM components or regulators, we found that four key circRNAs were generally positively and negatively correlated with TAAD overexpressed and underexpressed ECM-related genes, respectively (Supplementary Figure 1). CircUSP4 and circNOX4, which had higher AUC than the rest two key circRNAs, were more broadly related to ECM components and regulators, such as negatively correlated with collagen (COL6A6 and COL9A3) and fibrillin (FBN2) and positively correlated with MMPs (MMP1, 14 and 19) (Supplementary Figure 1). However, the above results were limited in gene expression level and further studies were needed.

Notably, the muscle function-related scores, including VSMC contraction and smooth muscle contraction, were enhanced in ceRNA network enrichment, while they were diminished in GSEA analysis in TAAD (Figures 4B, C). Since the ceRNA network was limited in the specific mechanism of circRNA-sponged miRNAs and individual genes, while GSEA results take the overall gene expression into consideration, the contradictory result of muscle function between ceRNA and GSEA illustrated that the key pathology of VSMCs dysfunction in TAAD (41–43) presumably resulted upon additional regulatory mechanisms of circRNAs. Spearman's correlation analysis also demonstrated the contradictory pattern of the key circRNAs with genes involved in positive regulation of smooth muscle contraction, of which the annotated circRNA target genes tended to be of positive correlation with the circRNAs, while others were opposite (Figure 4D). Additionally, the unannotated key circRNA, circNOX4, exhibited a similar pattern with the three other circRNAs (Figure 4D), highlighting the equivalent pathological status of the key circRNAs. Interestingly, circUSP3, which has the best diagnostic potentiality among the four circRNAs, focused on smooth muscle dysfunction and cell adhesion rather than inflammation according to correlation analysis (Figure 4C), suggesting that the destruction of aorta intrinsic components might be the hallmark and/or more pivotal in TAAD pathogenesis. Finally, the metabolic changes were observed with enhanced glycolysis and declined fat acid metabolism along with the circRNAs (Figure 4C). Hypoxia was found in TAAD, and HIF1α-induced glycolysis in macrophage was reported to promote inflammatory gene expression (44). The role of hypoxia in TAAD as a cause, result, or amplifier is still debatable.

### Correlation analysis of the key circRNAs with cell infiltration in TAAD

**Figure 5:**
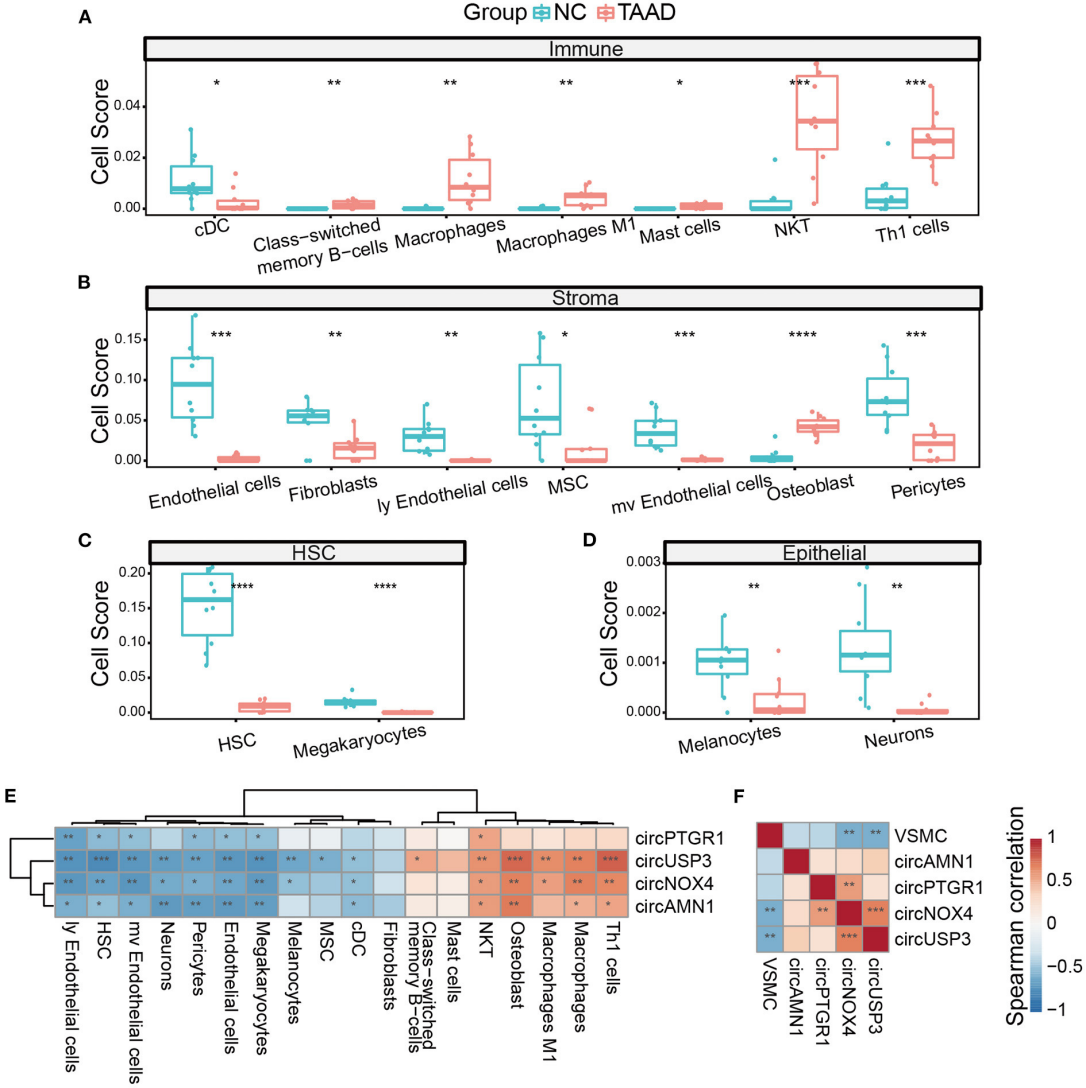
The circRNAs were correlated with the cell composition of TAAD. **(A–D)** Different cells, including immune cells **(A)**, stromal cells **(B)**, HSC **(C)**, and epithelial cells **(D)**, of TAAD aortic tissue and healthy control. **(E)** Heatmap of Spearman's correlation of circRNAs expression and different cell scores. **(F)** Heatmap of Spearman's correlation of circRNAs expression and VSMC score. *p < 0.05, **p < 0.01, ***p < 0.001, ****p < 0.0001 (Student's *t*-test and Spearman's correlation).

Cell composition alteration is still the key in tissue pathology. Although immune infiltration and cell apoptosis were reported in TAAD, the analysis of cell proportions and their relationship with circRNAs required more researches. Here, we utilized xCell (45), a novel gene signature-based method, to systematically portray the cellular heterogeneity landscape in TAAD tissue. Interestingly, the TAAD aorta was observed with pervasively increasing immune components and reduced stromal components (Figures 5A, B). The scores of NKT cells and Th1 cells were dramatically enriched in the TAAD aorta, and the scores of class-switched memory B-cells, macrophages (especially M1 macrophage), and mast cells were increased in TAAD as well, while the score of conventional dendritic cell (cDC) was reduced (Figure 5A). This result indicated that the cytotoxic effect and inflammation were enhanced in TAAD. As for stromal components, the loss of endothelial cells and fibroblasts was noticed in TAAD. Microvascular cell signatures, including microvascular endothelial cells and pericytes, were also decreased in TAAD (Figure 5B). Importantly, TAAD aortic tissue exhibited stemness loss characteristics, which were supported by the reduction of mesenchymal stem cells and hematopoietic stem cells (Figures 5B, C). Previous studies reported stemness signature loss in TAAD, and stem cells' potential contributed to aortic repair (46, 47). Incidentally, neurons and melanocytes were weakened as well in TAAD (Figure 5D), which supported the neuroactive ligand–receptor reduction in DEGs enrichment.

We further applied Spearman's correlation analysis of the key circRNAs and different cell infiltration. Notably, the key circRNAs demonstrated a dual vital role of positive correlation with immune cell scores and negative correlation with stromal cell scores, with circUSP3 showing the highest significance and broader effectiveness on almost all the different cell components, while circPTGR1 tending to be negatively related to the stromal cells (Figure 5E). Thus, the key circRNAs worked in a coordinative fashion. We further applied ssGSEA of VSMC single-cell marker genes since VSMC played a vital role in TAAD yet xCell software did not include VSMC calculation. Among the four key circRNAs, circNOX4 and circUSP3 were negatively correlated with VSMC score, indicating that these two circRNAs might participate in the loss of VSMCs (Figure 5F). Interestingly, the osteoblasts exhibited an increase in TAAD inflammation (Figure 5B) rather than osteoclasts, which was dissimilar from most common inflammations (48). Since xCell was based on cell markers, it was not rare to find the contrast expressions of individual marker genes. Therefore, we analyzed the expression of osteoblast markers and found three up-expressed markers (IBSP, GDF5, and PGK1) and three down-expressed markers (AMOTL2, SGCG, and EBF2) in TAAD, despite the outcome of the calculated score of osteoblast was positive. Notably, IBSP was reported as an activator of MMP2, which was the important metalloproteinase degrading ECM in TAAD (49, 50) while EBF2 inactivation led to bone mass loss and osteoclasts increasing. Thus, the osteoblast score in TAAD was complex and needed further study.

### The key circRNAs were sufficient to diagnose TAAD in plasma serum

**Figure 6:**
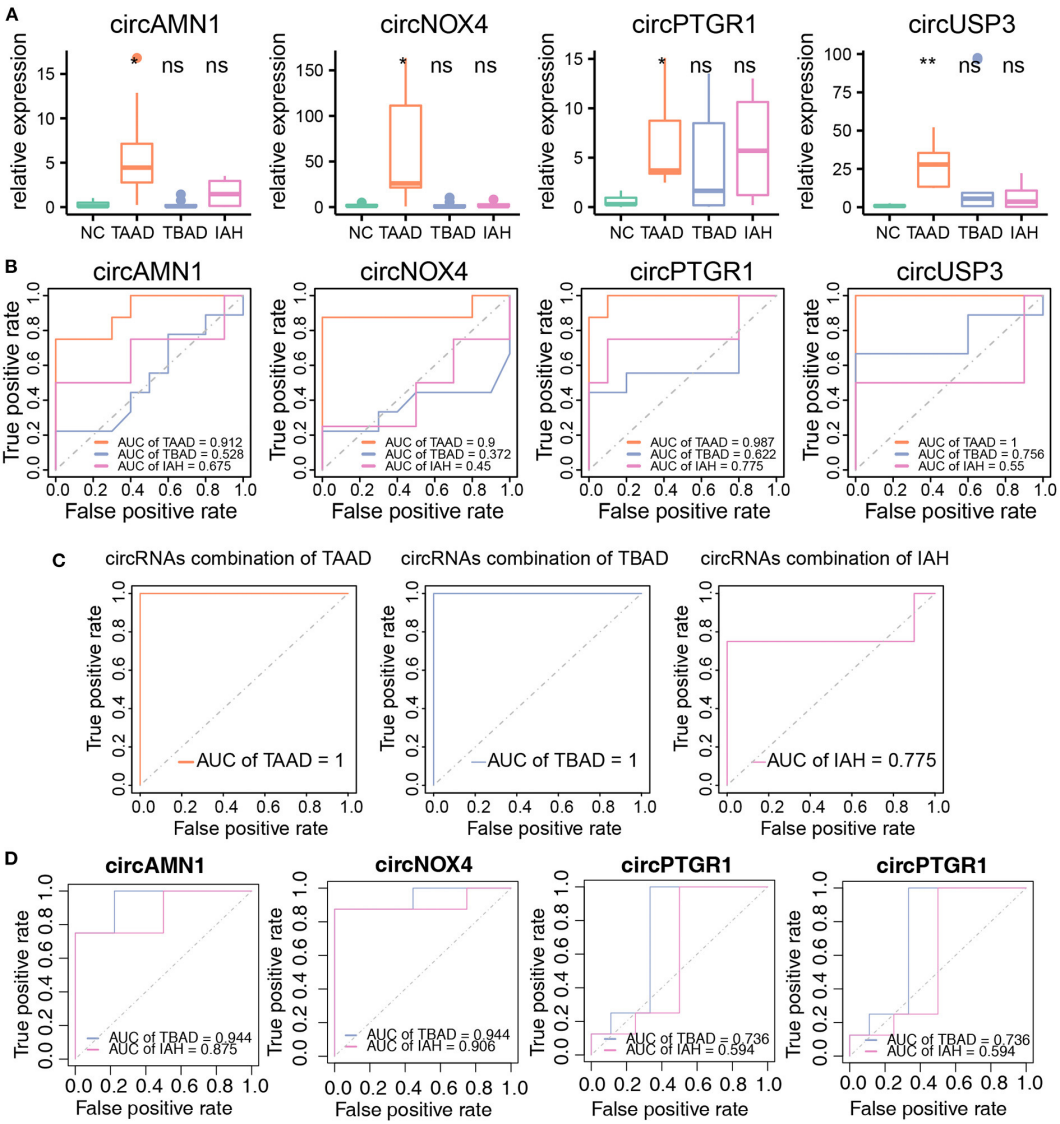
The circRNAs as biomarkers were efficient to diagnose TAAD in peripheral blood serum. **(A)** The circRNAs relative expression of NC (*n* = 10), TAAD (*n* = 8), TBAD (*n* = 9), and IAH (*n* = 4) in peripheral blood serum. Student's *t*-test was utilized to test different disease types compared to NC, respectively. **(B)** ROC curve analysis of the prognostic prediction efficiency of individual circRNA in TAAD, TBAD, and IAH from NC. **(C)** ROC curve analysis of the prognostic prediction efficiency of combination circRNAs in TAAD, TBAD, and IAH. **(D)** ROC curve analysis of the prognostic prediction efficiency of each circRNAs in TBAD and IAH from TAAD. *p < 0.05, **p < 0.01 (Student's *t*-test).

Finally, we assessed the diagnostic power of the key circRNAs in the peripheral blood by RT-PCR to evaluate the relative expression level of the circRNAs in the plasma serum in TAAD and healthy controls. As expected, the key circRNAs were all higher expressed in TAAD serum compared to those in the healthy controls, while they varied in TBAD and IAH (Figure 6A). As for the diagnostic efficiency of individual circRNA, all four key circRNAs exhibited solid diagnostic capabilities in distinguishing TAAD and healthy controls, but not in TBAD and IAH (Figure 6B). The AUCs of circAMN1, circNOX4, circPTGR1, and circUSP3 were 0.912, 0.9, 0.987, and 1, respectively (Figure 6B). Giving the limited sample size, it was worth noting that circUSP3 and circPTGR1 still had the diagnostic possibility of TBAD and IAH whose AUC was 0.756 and 0.775 for TBAD and IAH, respectively (Figure 6B). This result indicated that circUSP3 and circPTGR1 might be the shared molecular mechanism of TABD and IAH with TAAD, respectively, while circAMN1 and circNOX4 were more TAAD-related. The AUC of the four key circRNAs combined model was 1 in distinguishing TAAD and healthy controls (Figure 6C). Interestingly, the four combined circRNAs were also capable of classifying TBAD and health controls (AUC = 1) rather than IAH (AUC = 0.775) (Figure 6C). Considering that inflammation and compromised aortic integrity were the shared mechanisms of TAAD and TBAD, while the key circRNAs were highly related to the mechanism, it was believed that circRNAs were aortic dissection subtype dependent, and circUSP3 might be the common biomarker of TAAD and TBAD. Further studies were needed to confirm the hypothesis. Notably, circNOX4 and circAMN1 were equivalent to distinguish TAAD from TBAD whose AUCs both reached 0.944 (Figure 6D). As for discrimination of TAAD and IAH, circNOX4 also exhibited considerable specificity and sensitivity whose AUC was 0.906 while circAMN1 was less promising (AUC = 0.875) (Figure 6D).

In conclusion, the results verified that the four key circRNAs were adequate to diagnose TAAD in the peripheral plasma serum. However, determining the early onset of these circRNAs in the risk population is the key.

## Discussion

Circular RNAs were once regarded as nonfunctional products of aberrant RNA splicing due to their limited expression (51, 52). However, increasing evidence has certified that numerous circRNAs are abundant, functional, stable, and evolutionarily conserved in mammalian cells with the rapid advance in high-throughput deep sequencing technology and bioinformatics (9, 10, 53, 54). The stability and condition-dependent alternative expression of circRNAs endowed them as potential diagnostic biomarkers for various disorders (55–57). Moreover, various circRNAs have been suggested in diseases, acting as translational and/or transcriptional regulators *via* different mechanisms (11, 12, 52). Several cardiovascular diseases including cardiac hypertrophy and heart failure, atherosclerosis, aortic aneurysm, and thoracic aortic dissection, have been linked to circRNAs (58–60). The TAAD risk factors, including aging, hypertension, and inflammation, were reported to shift the circRNA expression profile (61–63). By the way, the four key circRNAs in this study were close to stem cell loss, indicating that these circRNAs might participate in the aging process. Furthermore, the altered expression of circRNAs is reported in TAAD, but the study of the pathological role of circRNAs is insufficient and requires a systematical elucidation.

In this study, we conducted an RNA-seq-data-oriented dissection of the circRNA profile in 10 pairs of patients with TAAD and healthy controls. A total of 26,566 circRNAs detected and identified by CIRCexplorer2 software, including 78 upregulated and 9 downregulated circRNAs, were harvested from patients with TAAD and controls. The lasso model identified four key circRNAs as the TAAD biomarkers, which fully discriminated the patients with TAAD from controls and were validated in the peripheral plasma serum, revealing the pathological relevance and diagnostic potential of circRNAs in TAAD.

Previously, circRNAs are believed irrelevant to their host gene expression (9). To assess this notion, we initially analyzed the DE circRNAs host genes by GO enrichment and KEGG pathway and found the association of these genes with the MAPK signaling pathway and adherens junction. The MAPK signaling pathway plays a pivotal role in directing diverse cellular processes including inflammation, cell stress response, proliferation, and apoptosis (64, 65). Mechanical stretch was reported to exacerbate rat aortic dissection by the activation of MAPK signaling pathway (66). Pathological disorganization of the adherens junction in endothelial cells was reported to be involved in aortic vascular intima injury, resulting in TAAD formation (67). Thus, the finding raised the possibility that the DE circRNAs' host genes might have aggravated TAAD. However, further evaluation of the expressions of MAPK signaling pathway and adherens junction genes resulted in no differences between TAAD and controls, indicating that the host genes of the DE circRNAs were unlikely involved in TAAD pathogenesis.

We then focused on the role of circRNAs in TAAD pathogenesis. Since miRNA sponging is the known general mechanism of circRNAs in gene regulation, which allows characterizing the function of the key circRNAs by constructing the circRNA-miRNA-mRNA network based on the online databases, we conducted this operation. Interestingly, the predicted target genes covered around two out of three (66.09%) of upregulated DEGs in the present dataset and featured the conventional pathological processes of TAAD, such as inflammation, ECM degradation, and aortic wall weakening, which were collaborated in TAAD pathogenesis. MiR-26b was previously reported to suppress TAAD development by directly targeting HMGA2 (68). Our analysis demonstrated that circPTGR1 was upstream of this mechanism and formed a more complete regulation chain of circPTGR1-miR-26b-HMGA2. The infiltrated inflammatory cells, including macrophages and various lymphocytes, are synchronized in high expression of proinflammatory factors (IL-6 and TNF) and MMPs (MMP1, 3, etc.), which are sensitive to inflammation signals and in turn induce ECM destruction and VSMCs apoptosis (26).

It is worth noting that, distinguishing from previous studies, we found that the TAAD upstream inflammatory cytokines were focused on IL-6 and IL-33 with massive downstream inflammatory responses linked to IFN-γ, TNF-α, IL-1, IL-2, IL-4, IL-6, IL-10, and IL-13 signaling, suggesting the uniqueness in triggering the inflammation of TAAD. IL-6 is a well-known pan-inflammatory factor coordinating the initiation of tissue inflammation, which is upregulated in various disorder conditions and highly related to TAAD (69, 70). Importantly, IL-33 is involved in various immune cells activation (Th2 cells, natural killer cells, etc.) and amplifies the immune responses under the induction of mechanical stress and inflammation (71, 72). Vascular weakening in TAAD also exhibited an interesting signature. The fibroblast score was reduced in TAAD rather than the smooth muscle score. On the contrary, the dysfunction of muscle contraction illustrated by the loss of neuroactive ligand–receptor interaction and massive downregulation of various voltage-gated channels was observed in TAAD, highlighting the phenotype switch in VSCMs of TAAD (41, 42, 73).

The GSEA based on the correlation coefficients of circRNAs and gene expression demonstrated an essentially identical pathological effect of the key circRNAs with the enhancement of inflammation response and P53 signaling pathway, and the alteration and weakening of ECM, which were positively related to the key circRNAs, except the muscle function-related scores, including VSMC contraction and smooth muscle contraction, which were diminished in GSEA analysis in TAAD (Figure 4B, C) while they were enhanced in ceRNA network-based enrichment. The contradictory results regarding muscle functions between ceRNA analysis and GSEA illustrated a key difference in the pathology of VSMCs dysfunction in TAAD (41, 42) and presumably resulted in additional regulatory mechanisms of circRNAs other than miRNA sponging.

Finally, we correlated the cell composition of TAAD tissues with the key circRNAs. We found that circNOX4, circAMN1, and circUSP3 were pervasively related to the infiltration of inflammatory cells and loss of stromal components, while circPTGR1 was negatively related to the stromal cell components. These results argued that the key circRNAs played an essential dual role in inflammation promotion by facilitating inflammation cell infiltration and ECM degradation by stromal components (vascular proper cells) loss *via* interacting with miRNAs, which accelerated the TAAD development. As discussed earlier, circUSP3 preferred to involve in smooth muscle function and cell adhesion in addition to other classical molecular signatures of TAAD pathology, which was not reflected in cell component analysis, indicating that the dysfunction of intrinsic aorta components was also the key process of TAAD. VSMC was reported as the major source of ECM composition, and impairment of these cells resulted in intolerance of mechanical stress of high blood pressure in large vessels (73). As for the cell proportion, circUSP3 was correlated with almost all different cell compositions. The mismatch of circUSP3 in cellular and molecular annotation needs further research. In summary, GO annotation, KEGG pathway, GSEA, and cell composition analysis indicated that the key circRNAs in TAAD might involve in the pathologic process of TAAD. However, further studies are necessary to focus on these circRNAs including their potential-related miRNAs and mRNAs and target cells.

## Conclusion

Our results emphasize the crucial position of circRNAs in TAAD, especially as a superior regulator of pathological molecules, and their potential as diagnostic biomarkers. In the current study, according to the bioinformatic analyses of RNA-seq data, four key DE circRNAs were selected, which were all significantly upregulated in the TAAD dataset and validated in clinical samples. Furthermore, ROC analysis suggested that all four key circRNAs could potentially serve as circulating diagnostic biomarkers of TAAD. Target miRNA prediction analysis demonstrated that these circRNAs contain numerous miRNA-binding sites that may be involved in inflammation, ECM degradation, cell adhesion, and junction. Studies have shown that the alteration of ECM and inflammation cascade were the risk factors of aortic dissection. Furthermore, cell composition analysis found that all four key circRNAs were pervasively correlated to immune cell infiltration and stromal loss. Collectively, these results demonstrated the vital role of the selected circRNAs in the pathogenesis of TAAD. However, the precise mechanism of these circRNAs including the exact ceRNA regulation network and the cell specificity in TAAD is still indistinct. It is necessary to integrate the functional and mechanistic roles of these circRNAs in subsequent studies.

## Data availability statement

Publicly available datasets were analyzed in this study. This data can be found here: https://www.ncbi.nlm.nih.gov/geo/query/acc.cgi?acc=GSE153434. The generated circRNA expression matrix and code are available by request to the corresponding authors.

## Ethics statement

The studies involving human participants were reviewed and approved by the Medical Ethics Committee of Nanjing Drum Tower Hospital, the Affiliated Hospital of Nanjing University Medical School (Institutional Review Board File 2016-152-01). The patients/participants provided their written informed consent to participate in this study.

## Author contributions

QL and QG designed the study and wrote the manuscript. QL ran the bioinformatics analyses. ZZ, QT, and YW performed the experiment. HL, AL, JX, YW, HM, LW, SS, and DW provided clinical specimens and data, technical support, and conceptual advice. All authors contributed to the article and approved the submitted version.

## Supplementary material

Supplementary Figure 1 Heatmap of Spearman's correlation of four key circRNAs with ECM-related DEGs. Each condition of DEG was annotated in the column. ^*^*p* < 0.05, ^**^*p* < 0.01, ^***^*p* < 0.001.
