# Supplementary figures and images for: Identification of pathological-related and diagnostic potential circular RNAs in Stanford type A aortic dissection
Source: Front Cardiovasc Med. 2023 Jan 13;9:1074835. doi: 10.3389/fcvm.2022.1074835 (PMC9880160; doi:10.3389/fcvm.2022.1074835)

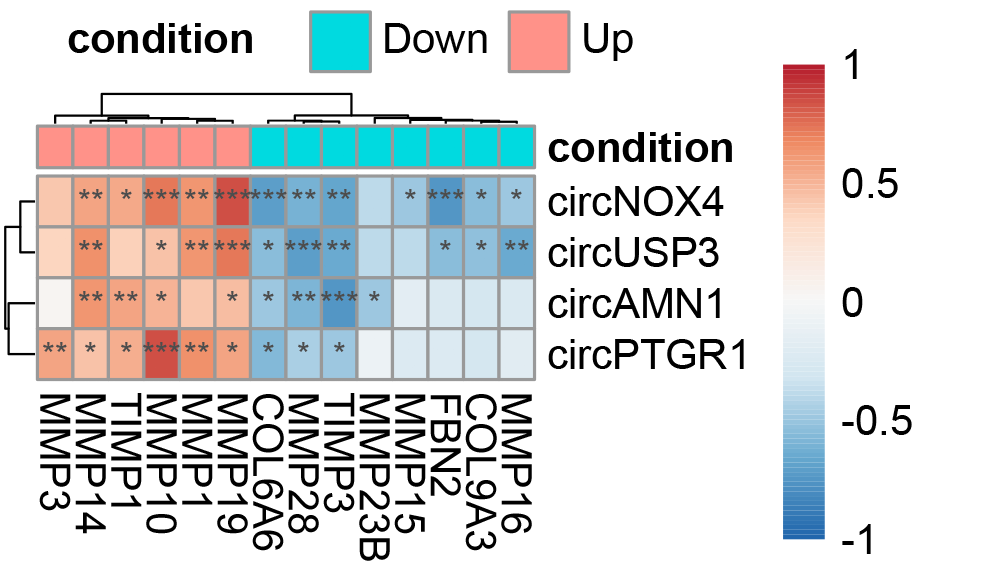

Supplement: Supplementary Figure 1 — Heatmap of Spearman's correlation of four key circRNAs with ECM-related DEGs. Each condition of DEG was annotated in the column. *p < 0.05, **p < 0.01, ***p < 0.001. [file Image_1.TIF]
